# RNAi therapy to the wall of arteries and veins: anatomical, physiologic, and pharmacological considerations

**DOI:** 10.1186/s12967-017-1270-0

**Published:** 2017-07-28

**Authors:** Christoph S. Nabzdyk, Leena Pradhan-Nabzdyk, Frank W. LoGerfo

**Affiliations:** 1 Department of Anesthesiology, Perioperative and Pain Medicine, Brigham and Women’s Hospital, Harvard Medical School, Boston, MA USA; 2 Frank W. LoGerfo Division of Vascular and Endovascular Surgery, Beth Israel Deaconess Medical Center, Harvard Medical School, 110 Francis Street, Boston, MA 02215 USA

## Abstract

**Background:**

Cardiovascular disease remains a major health care challenge. The knowledge about the underlying mechanisms of the respective vascular disease etiologies has greatly expanded over the last decades. This includes the contribution of microRNAs, endogenous non-coding RNA molecules, known to vastly influence gene expression. In addition, short interference RNA has been established as a mechanism to temporarily affect gene expression. This review discusses challenges relating to the design of a RNA interference therapy strategy for the modulation of vascular disease. Despite advances in medical and surgical therapies, atherosclerosis (ATH), aortic aneurysms (AA) are still associated with high morbidity and mortality. In addition, intimal hyperplasia (IH) remains a leading cause of late vein and prosthetic bypass graft failure. Pathomechanisms of all three entities include activation of endothelial cells (EC) and dedifferentiation of vascular smooth muscle cells (VSMC). RNA interference represents a promising technology that may be utilized to silence genes contributing to ATH, AA or IH. Successful RNAi delivery to the vessel wall faces multiple obstacles. These include the challenge of cell specific, targeted delivery of RNAi, anatomical barriers such as basal membrane, elastic laminae in arterial walls, multiple layers of VSMC, as well as adventitial tissues. Another major decision point is the route of delivery and potential methods of transfection. A plethora of transfection reagents and adjuncts have been described with varying efficacies and side effects. Timing and duration of RNAi therapy as well as target gene choice are further relevant aspects that need to be addressed in a temporo-spatial fashion.

**Conclusions:**

While multiple preclinical studies reported encouraging results of RNAi delivery to the vascular wall, it remains to be seen if a single target can be sufficient to the achieve clinically desirable changes in the injured vascular wall in humans. It might be necessary to achieve simultaneous and/or sequential silencing of multiple, synergistically acting target genes. Some advances in cell specific RNAi delivery have been made, but a reliable vascular cell specific transfection strategy is still missing. Also, off-target effects of RNAi and unwanted effects of transfection agents on gene expression are challenges to be addressed. Close collaborative efforts between clinicians, geneticists, biologists, and chemical and medical engineers will be needed to provide tailored therapeutics for the various types of vascular diseases.

## MicroRNA (miRNA) and short interfering RNA (siRNA)

Short interfering RNA and miRNA are useful tools to temporarily suppress target genes. Different from siRNA, miRNA are endogenously occurring short RNAs heavily involved in regulating numerous cellular functions. A recent review provides a more in-depth comparison of siRNA and miRNA, including respective transfection modalities [1].

While siRNA usually is used to silence one specific gene, a single miRNA has the ability to affect expression of multiple genes. Approximately one-third of the genes are regulated by miRNA and it has been shown that several miRNA are able to bind to the identical 3′UTR region [2]. Various reviews summarized how different miRNA clusters affect vascular biology [3, 4]. miRNA are small non-coding RNA molecules (~22 bp) that contain an imperfectly base-paired hairpin segment [5]. In contrast, siRNA are mostly exogenous and while similar in length, siRNA form perfectly complementary double-strand structures [5]. After Drosha and Dicer mediated maturation, single-stranded miRNA enters the RNA-induced silencing complex (RISC). Subsequently this complex directs the miRNA to the target mRNA resulting its translational repression [3, 4]. Additionally, miRNA have the ability to regulate gene transcription after their nuclear import [6]. Similar to miRNA, siRNA hybridizes with its target mRNA in RISC leading to its catalytic degradation. We recently reviewed current siRNA targets for ATH, AA and IH and thus will focus here primarily on aspects of RNAi delivery to the vascular wall [7, 8].

### Anatomical and physiological differences of vascular conduits and disease

The anatomy of arteries and veins reveals similarities yet also significant histological differences. The inner lining of arteries and veins (tunica intima) consists of a single layer of EC seated on a basement membrane (BM). The layers of the BM are composed of an intricate network consisting of various collagens types, glycoproteins and proteoglycans, and cell–cell, and cell–matrix interaction regulators such as integrins. Different from sinuses in the liver and spleen, which have a discontinuous BM and therefore promote extravasation of RNAi, the BM of large arteries and veins is continuous and provides an anatomical barrier. The BM is connected to a thin layer of subendothelial connective tissue. Different from veins, arteries and arterioles also have as the outer margin of the tunica intima a prominent internal elastic membrane (lamina) with elastin containing fibers. This layer presents another physical barrier for RNAi for transmural delivery.

The next layer, tunica media is mostly comprised of circularly arranged smooth muscle cells with interspersed reticular and elastic fibers. This layer’s thickness varies significantly between veins and arteries. Naturally a thicker layer of SMC in larger arteries provides a greater challenge for a homogenous, transmural transfection when compared to a thin-walled vein graft. As mentioned before, VSMC appear less susceptible to RNAi compared to EC [9–12]. If VSMC are the designated primary target cells and in the absence of a technology to achieve VSMC-specific delivery, it is possible that a disproportionate amount of RNAi oligomers will transfect nearby EC. This needs to be factored into the silencing strategy as it could cause deleterious effects. This is especially the case if regulators of apoptosis, cell cycle, or migration are targeted [13–15].

The outer most layer, tunica adventitia is made up of connective tissue. In large arteries an external elastic membrane may separate the tunica adventitia from the tunica media. Large arteries and veins receive additional blood supply via vasa vasorum, which enter from the adventitial site. Given the low oxygen content of venous blood, it is conceivable that large veins are more dependent on vasa vasorum for their nutrient/oxygen supply than arteries. In the setting of vein graft implantation, that supply route may be disrupted. This may contribute to the formation of IH. It is also believed that vasa vasorum play a significant role in atherosclerosis progression [16].

Interestingly, vasa vasorum appear to cease to supply oxygenated blood to the arterial wall at the level of the renal arteries, which may help explain an increased incidence of aortic aneurysms in this region [17]. The expression of endothelial alpha(v)beta3 integrin was used to pharmacologically target vasa vasorum and thus modulate atherosclerosis [16].

### Atherosclerosis

Atherosclerosis (ATH) by some is considered a chronic inflammatory condition that involves a complex interplay of immune and stromal cells, cytokines and enzymes. It is characterized by a disturbed loco-regional lipid metabolism, extracellular matrix organization, and cellular homeostasis leading to lipid plaque build-up and ultimately risk for plaque rupture and vascular narrowing or occlusion [8, 18]. Hyperglycemia is further believed to induce VSMC resistance to apoptosis and thus contributing to diabetic vasculopathy [19]. Multiple potential targets for a siRNA therapy of ATH, AA, and IH have been identified, yet clinically convincing evidence of an efficacious therapy is lacking [7, 8].

A recent review from Welten et al. dissects the contribution of various miRNA clusters to the maladaptive processes of atherosclerosis and restenosis [20]. Prominent miRNA amongst those are miR-126, miR-155, the miRNA gene clusters 17-92, and miRNA 23/24/27, 143/145 [20]. Additionally, transcoronary gradients of anti-atherosclerotic miR-126-3p and miR-145-5p were found to correlate with the extent of thin cap fibroatheromas [21]. miRNA-210 is believed to enhance fibrous cap stability in atherosclerotic lesions [22]. miRNA targets for atherosclerosis were also recently reviewed elsewhere [23].

### Intimal hyperplasia (IH)

In the case of IH in the setting of vein graft surgery and vascular implantation injury, synthetic VSMC migrate towards the lumen of the vessel. There, VSMC proliferate and secrete pro-inflammatory cytokines and extracellular matrix proteins forming the bulk of the eventual IH lesion [7].

miRNA have also shown to be involved in IH formation [24, 25]. One of the genes that has been shown to regulate the expression of multiple miRNA in VSMC is Thrombospondin 1 (TSP-1), a glycoprotein involved in cell–cell and cell–matrix contacts [25]. The most upregulated miRNA in response to TSP-1 was miR-512-3p, while the most downregulated miRNA was miR-25-5p. Interestingly, five members of the mir-17-92 cluster were downregulated [25].

Some strategies to attenuate IH aimed at attenuating VSMC proliferation after vascular injury [13, 26, 27]. While this seems to make physiologic sense, clinically the initial results have been disappointing. The largest, prospective, randomized controlled trial in humans to date (PREVENT III) designed to attenuate vein graft failure by use of oligonucleotide anti-sense therapy targeting E2F, a transcription factor promoting G1/M transition, failed to show protection from IH [26, 27]. There has been a lot of debate as to why to the PREVENT III trial failed. Be it the degree or duration of gene silencing, or the target E2F itself. Another consideration is that EC are more susceptible to RNAi than VSMC under identical conditions [9–12]. Hence, it is conceivable, that bystander EC were transfected and their altered ability to proliferate in the setting of vascular injury may have contributed to the trial’s failure.

## Anatomical proximity of EC and VSMC

Given the immediate proximity of EC and VSMC in the vascular bed, it appears a rather difficult task to deliver RNAi exclusively to one of the two cell fractions. In addition, particularly the arterial vessel wall consists of a thick muscular layer with multiple layers of VSMC stacked on top of each other. In order to achieve a transfection success across all VSMC layers, a high dose of RNAi oligomers as well as potent transfection agents [e.g. liposomal or cationic polymer based such as polyethyleneimine (PEI)] may have to be used [9, 28, 29]. This again may result in inadvertent transfection of other cells residing in the vascular wall including EC, pericytes, dendritic cells and fibroblasts. These cells have important roles in vascular homeostasis and in orchestrating the response to injury. Furthermore, the aforementioned transfection agents themselves elicit profound effects on the transcriptome of vascular cells thus additionally complicating the situation [29].

### Distinct gene expression and stress response patterns of VSMC and EC from different vascular beds

Not surprisingly, relevant differences exist amongst the same cell type (EC or VSMC) within the separate vascular beds. A study comparing canine VSMCs within the aorta (Ao), branch pulmonary artery (bPA), main pulmonary artery (mPA) and inferior vena cava (IVC) revealed not only obvious histologic differences in layer thickness and cellular organization, but also significant protein expression differences with regards to smooth muscle actin (SMA), myosin heavy chain (MHC) and smooth muscle myosin heavy chain isoform 2 (SM2) [30].

Separately, differential expression patterns were found with regards to Hox4. Products of the hox gene family regulate the cranio-caudad organization of the body during embryogenesis. Differential Hox4 expression has been found along the aorta in EC and VSMC of baboons. Thoracic EC and VSMC revealed higher Hox4 levels compared to corresponding abdominal aortic cells [31]. Further, human AAA specimens were found to have a significantly lower Hox4 expression when compared to healthy controls [31].

Another example is the protein testin, which was found to be differentially expressed in coronary and internal mammary arteries. Testin is a cytoskeleton-associated protein that localizes along actin stress fibers, at cell–cell-contact areas, and at focal adhesion plaques. Testin was also found to have tumor suppressor gene function and may be relevant for EC function [32, 33]. Testin silencing in EC promoted oxidized-LDL-mediated monocyte adhesion to ECs, EC migration and the transendothelial migration of monocytes, while overexpression of testin mitigated these effects [33].

Aside from varying intrinsic protein expression profiles it could be shown that VSMC from different segments of the circulation respond distinctly to noxious stimuli. Oxidized low-density lipoprotein (OxLDL) treatment in saphenous vein graft VSMC (SV-VSMC) led to an increase in proliferation and migration as well as NF-kappa B activation. In contrast, OxLDL inhibited proliferation and migration in coronary VSMC. In addition, significant differences in cytokine, chemokine extracellular matrix protein expression were noted between these cell groups [34].

In line with this are observations derived from in vitro experiments, which revealed that SV-VSMC were found to be significantly more proliferative and displayed a more migratory and invasive behavior than internal mammary artery VSMC [35].

Yet in contrast, human VSMC derived from atherosclerotic lesions of infragenicular arteries displayed significantly increased rates of proliferation, adhesion, and migration as compared to human saphenous vein VSMC [36].

Also, the response to hypoxia appears to be different in arterial and venous SMC. While both cell fractions showed a decreased proliferative response to hypoxia, arterial SMC showed a significant upregulation of vascular endothelial growth factor (VEGF-A), while venous SMC did not. VEGF-R 2 expression was found to be upregulated in hypoxic venous SMC, but not in arterial SMC [37]. The same investigators hypothesized that VEGF-A may be a target gene of mir-125b, 29a, and 29b. Interestingly, hypoxic conditions induced a 15-fold increase of mir-125b in VSMC compared with ASMC, which did not show a significant change. In the author’s opinion this could explain the lack of VEGF-A upregulation in hypoxic venous SMC [37]. Other relevant miRNA include miR-143 and 145, which appear to have significant roles in SMC fate and plasticity [38, 39]. Adding complexity to the VSMC-EC interaction is the existence of intercellular nanotubes that shuttle VSMC derived miR-143 and 145 to EC and thereby influence EC biology [40]. This mechanism, at least in part, was triggered by TGFβ and vessel stress [40].

Further complicating is the finding that significant differences between EC from various vascular beds exist [41]. There appear to be even differences in the phenotypes of aortic valve EC on the ventricular site when compared to the aortic site. In vitro shear-stress experiments revealed that waveforms simulating the ventricular flow pattern selectively upregulated the atheroprotective transcription factor Kruppel-like factor 2 (KLF2), while suppressing the pro-inflammatory chemokine monocyte-chemotactic protein-1 (MCP-1) in EC [42].

Separately, bone morphogenetic protein (BMP) signaling has been found relevant for a variety of pathways involved in EC biology. Heterogeneous transcriptional activity seemed to correlate with vasculature undergoing hemodynamic alterations [43]. BMP signaling is connected to other major signaling pathways including Notch, WNT, and is altered in response to hypoxia [44].

Locoregional differences in miRNA expression patterns as a function of flow disturbances have been investigated, coining the term “flow-sensitive miRNA” or “mechano-miRNA” [45]. miRNAs such as, miR-10a, miR-19a, miR-23b, miR-17-92, miR-21, miR-663, miR-92a, miR-143/145, miR-101, miR-126, miR-712, miR-205, and miR-155 are counted amongst those mechano-miRs [45].

In addition, there appear to be differences in siRNA susceptibility between EC and VSMC and possibly even between the various VSMC fractions under identical transfection conditions [9, 10].

All these biological differences help explain the varying loco-regional responses to vascular injury including atherosclerosis, postangioplasty restenosis and vein graft disease [34]. These data underscore the importance of a comprehensive characterization of the specific target cell subtype and the spatial differences with the cell subtype. Further, it is important to understand the pathophysiologic consequences that a specific intervention inflicts on these cells. This includes the effects of RNAi therapy itself including the effects of the transfection reagents used.

### RNAi delivery methods

Systemic, naked siRNA administration without technology to target specific cells types leads to renal siRNA secretion within 20 min as well as rapid plasma nuclease degradation. In addition, significant degradation and phagocytosis via the reticuloendothelial system occurs which also shortens the siRNA’s systemic half-life [46].

In parallel, several chemical modifications to the RNA have been introduced including locked nucleic acid technology (LNA), in which an additional covalent bond within the RNA backbone is formed. This has proven to improve stability to the RNA molecule and protection from phagocytosis [47, 48].

Liposomes are spheres consisting of customizable phospholipid layers that can be used for drug delivery. Liposomes widely used to facilitate diagnostic and experimental siRNA silencing vitro and are one of the most commonly used transfection modality [9, 49, 50]. However, liposomal siRNA formulations also undergo significant uptake by macrophages, liver and spleen (RES) limiting the ability to successfully reach the target genes in vivo. In addition, problems with toxicity, immune response, non-specific uptake, and the risk of off-side effects have been raised [9, 49, 51, 52].

The introduction of poly(ethylene glycol) (PEGylation) into liposomal formulations is another commonly performed process aimed at improving drug delivery efficacy. PEGylation is thought to confer some protection from mononuclear phagocytosis through the creation of a protective hydrophilic film on the surface of the liposome and decrease renal clearance due to a size increase of the liposomal complex [53–57].

Cationic polymers such as polyethyleneimine (PEI) can also be used to complex RNA complexes and improve transfection efficiency into vascular cells in vitro and in vivo [28, 58–60]. The positive surface charge of PEI-siRNA complexes may be utilized for adsorption onto vascular materials [58]. Incorporation of albumin into PEI-siRNA complexes provided protection from extracellular endonucleases and improved internalization and silencing efficiency in vitro [61]. However, PEI has been shown to have significant impact on global gene expression, leading to changes in 213 genes in human aortic SMC. These genes were mostly related to inflammation and immune response. These findings illustrate how PEI alone could affect the results of the RNAi therapy [29].

Another promising modality is RNAi containing nanoparticles. Some of these nanoparticles have self-assembling properties. The individual composition of these nanoparticles is highly customizable and can be adjusted to the specific needs of the therapy approach [62–66]. These nanoparticles can consist of degradable, biologically compatible materials such as poly(lactide-c-glycolide) (PLGA) [67]. The polymer’s degradation profile might allow for sustained RNAi release for vascular delivery [68, 69]. Polymer blends and co-polymers may also facilitate sequential RNAi release.

RNAi encoded in lentiviral and adenoviral vectors was successfully delivered to cardiovascular cells and other tissues [13, 65, 70–74]. Due to concerns of immunogenicity associated with older adenoviral constructs and possible insertional mutagenesis by lentiviruses, novel adenovirus associated viruses (AAV) have been developed. AAV have shown some promise for anti-angiogenic therapies for human retinal diseases [75–77]. While AAV is a powerful tool, there is ongoing research to develop modified AAV surface capsids for cell-specific RNAi delivery [78].

Many reviews exist that provide comprehensive overviews of RNAi vectors, chemical modifications and transfection agents including detailed capabilities and current limitations of either technology [50, 79, 80]. For the purpose of this review we will focus on approaches pertaining to vascular RNAi delivery.

### Results of vascular RNAi therapy for vascular injury remodeling

#### Local intraluminal RNAi delivery

Depending on the target cell (EC versus VSMC), local intraluminal RNAi delivery represents a logical and simple approach. Obvious challenges are the interaction of RNAi and its delivery vehicles with the components of blood, penetration in the cellular and non-cellular layers of the vessel wall, and washout from the site of delivery.

Single application can be accomplished via injection with or without distal occlusion or possibly from balloon catheters. In the setting of vein bypass grafting the RNAi solution may be infused into the graft. Similarly, brief distal arterial occlusion and target vessel distention is feasible in certain settings and may be used to enhance RNAi delivery.

Utilizing above approach PEI complexed TSP-2-siRNA was successfully administered to the denuded wall of rat carotid arteries and yielded a lasting silencing effect. While this resulted in desirable changes in transforming growth factor–beta (TGF-β) and matrix metalloproteinase-9 (MMP-9) signaling, as well as anti-inflammatory M2 macrophage polarization, it did not affect intima/media ratios [59].

In contrast, intraluminal siRNA targeting transcription factor activation transcription factor-4 (ATF-4), a downstream target of the mitogen fibroblast growth factor-2 (FGF-2) in balloon injury rat carotid arteries did lead to a measurable decrease in intimal hyperplasia [81].

Likewise, adenovirus mediated silencing of A disintegrin and metalloproteinase with thrombospondin motifs-7 (ADAMTS-7), a metalloproteinase reduced intimal VSMC proliferation in a rat model of balloon-induced vascular injury [82].

Several studies involving miR expression modulation in injured vascular walls have yielded some encouraging results. Amongst others, miR- 24 and miR-29b were found to be decreased in balloon-injured rat carotid arteries [73, 83]. MiR-24 delivery to the carotid artery wall decreased IH possibly through inhibiting Wnt4 signaling [73]. Local miR-29b delivery decreased IH formation possibly via downregulation of MMP-2 and myeloid leukemia cell differentiation MCL-1, a known inhibitor of apoptosis [83]. Similarly, delivery of miR-34c to the rat carotid artery after catheter injury has shown to attenuate stem cell factor expression and subsequently mitigated formation of IH [84]. MiR-132 delivery also mitigated IH via inhibition of VSMC proliferation in a carotid artery injury model [85]. However, thus far it has been difficult to translate findings of IH reduction from rodent models into large animal models or even human studies. This may be in part due to the thicker muscular vascular wall, limiting a homogenous transmural cell transfection in larger animals as well as possible differences in the individual species’ cell biology.

Sustained, transluminal, local delivery has been attempted by using RNAi coated, implanted stents/stent grafts. Plasmid DNA has been successfully transferred to the arterial wall in a pig stent-angioplasty model [86]. In line with this work, layer-by-layer (LBL) technology has emerged as an effective way to deposit multiple thin films of coating on surfaces and has shown to be one way to enhance sustained oligonucleotide delivery to the vascular wall from stent surfaces in vitro [87, 88]. Likewise, LBL technology utilizing siRNA nanoparticles allowed for sustained ex vivo siRNA transfer to the porcine arterial wall [89].

Alternatively, various biodegradable coatings such as poly(d,l-lactic-co-glycolic acid) (PLGA) and poly (l-lactide) (PLLA) are FDA approved as stent coatings that potentially could provide a sustained release RNAi to the vessel wall [90–93]. These materials are biocompatible and predictably degrade fully over time.

#### Local perivascular RNAi delivery

Perivascular delivery may have advantages over luminal administration in that the RNAi delivery vehicle is not exposed to blood and systemic arterial pressures. This could have benefits with regards to sustained delivery. Vasa vasora of large arteries and veins may aid in the perivascular delivery. Unwanted washout to other regions of the circulation may also be less. Further, if VSMC are primarily targeted, a perivascular approach may limit EC exposure to RNAi and its vehicle/vector.

Several studies have shown that perivascular delivery of RNAi is feasible and can result in measurable histologic effects with regards to IH formation. Similar to intraluminal delivery, ADAMTS-7 (a disintegrin and metalloproteinase with thrombospondin motifs 7) silencing after perivascular delivery of ADAMTS-7 siRNA in pluronic gel mitigated IH in injured rat arteries [82, 94].

Silencing of STAT-3, a transcription factor of multiple receptors also inhibited VSMC and decreased IH via expression of Bcl-2 and cyclin D1 in rat vein grafts without increases in apoptosis. siRNA was encapsulated with the liposomal transfection reagent Lipofectamine and deployed in a “Bioprotein gel” that was applied onto the adventitia of the rat vein grafts [95].

Likewise, siRNA targeting the heparin-binding growth factor midkine (MK) mixed with atelocollagen was administrated to the external wall of rat vein grafts. MK silencing decreased immune cell recruitment, and cell proliferation within MK siRNA-treated vein grafts [96].

#### Systemic RNAi delivery

Primary systemic delivery requires large doses of RNAi and its delivery vehicle thus increases cost, and the chance of off-site effects and toxicity.

In a mouse model of carotid injury the transcription factor GATA-2 was found to be reduced. GATA-2 regulates multiple EC specific genes as well as miR-126 and miR-221. Systemic miR-126-coupled nanoparticles enhanced miR-126 availability in the carotid artery and improved re-endothelialization of injured carotid arteries in vivo [97].

Independently, miR-181b has been shown to inhibit TNF-α induced downstream NF-κB signaling. Including vascular cell adhesion protein 1 (VCAM-1), intercellular adhesion molecule-1 (ICAM-1), E-selectin, and tissue factor. In a rodent model of photochemical injury-induced carotid artery thrombosis, systemic miR-181b delivery conferred some protection from thrombin-induced EC activation and arterial thrombosis [98].

#### Cell specific siRNA delivery

The endothelial cell biology has been well studied and various EC specific (CD31, TIE2, ICAM-1, VCAM-1, E-selectin) cell surface markers have been described in the literature as targets to provide a more directed, cell specific RNAi therapy [99–103].

Recent results showed that cationic lipoplexes can be designed that result in a predominant uptake into the vascular endothelium and thereby minimizing offsite-target effects [102]. Further work yielded a formulation that provided preferential pulmonary endothelial uptake [104].

While activated, VCAM-1 expressing EC were successfully targeted by PEGylated antibody-targeted lipoplexes in vitro. However, these promising results did not result in successful silencing in vivo [101].

While not exclusive, chemokine receptor CXCR-4 is constitutively expressed in EC. Peptide carriers as RNAi vehicles that contain a CXCR-4 ligand increased efficacy of VEGF-siRNA to EC [105].

Another intriguing approach resulted from data of an in vivo phage display in the setting of a partial carotid ligation model of flow-induced atherosclerosis in mice [100].

This led to the identification of two peptides (CLIRRTSIC and CPRRSHPIC) that specifically bind to EC exposed to turbulent flow in pro-atherogenic regions. These peptides were conjugated to polyethylenimine (PEI)-polyethylene glycol (PEG) to generate polyplexes containing ICAM-1 siRNA. CLIRRTSIC polyplexes carrying si-ICAM-1 specifically bound to mouse endothelium and silenced ICAM-1 expression in disturbed flow regions in a mouse model [100].

It remains to be seen if successful transfection of a rodent arterial wall can be translated into larger animal studies or even humans given the significant differences in wall thickness. Even if a similar transmural transfection result is achieved, it may come at the risk of higher toxicity and off-site effects given as larger doses of transfection agents and RNAi oligonucleotides may be required.

### Target choice

Vascular injury is characterized by a dramatic upregulation of thousands of genes within a matter of hours as demonstrated in a study of vein graft implantation in canine [106]. Over the following weeks these gene expression changes largely approach baseline [106]. Despite this apparent gene expression “near-normalization” after 4 weeks, the process of vascular remodeling and thus the growth of vascular lesions continues past that point. It is therefore of interest to have the option of a sustained release of RNAi to the vascular wall to change gene expression past the immediate period of graft implantation or endovascular intervention. Analysis of gene networks revealed significant signaling redundancy, which suggests it might be prudent to silence multiple genes simultaneously and/or sequentially in order to optimize the clinical effect of siRNA therapy. This became evident in a recent rat carotid artery angioplasty model, in which the extracellular matrix protein thrombospondin-2 was silenced through single intravascular siRNA administration. Anti-TSP-2 siRNA complexed with PEI resulted in TSP-2 protein level suppression for at least 21 days. This resulted in changes in downstream transforming growth factor–beta (TGF-β) and matrix metalloproteinase-9 (MMP-9) signaling and increased M2 macrophage polarization. However, no significant changes in the intima/media ratios were noted [59]. This data underscores the importance of target gene selection. While in this case, TSP-2 silencing yielded the anticipated downstream signaling effect and even a desirable macrophage phenotype switch, it did not result in a histologically relevant effect.

Given the discussed issues of cell specific RNAi delivery, target gene choice becomes a conundrum. Silencing of the target gene should result in a robust alteration in the biology of the targeted cell. In contrast, inadvertent target gene silencing in bystander cells should not impede those cells’ inherent biological activities. For example, the cell cycle and apoptosis regulator survivin (SVV) is highly upregulated in SV-VSMC the setting of IH [13, 107]. This appears to promote the proliferative, apoptosis resistant and migratory VSMC phenotype responsible for IH. In vitro silencing of SVV in SV-VSMC resulted in a cell cycle block with a subsequent decrease in proliferation. While it did not increase the rate of apoptosis in SV-VSMC to noxious stimuli, it did impair SV-VSMC migration.

In contrast, it has been shown, that overexpression of SVV in EC resulted in increased viability and migratory capability, but reduced apoptosis. SVV overexpressing EC were also associated with higher levels of angiogenesis in vivo [13, 107–110].

One of those candidate genes that differentially affects biology of EC and VSMC may be MARCKS (myristoylated alanine rich protein kinase C substrate) [111–114]. MARCKS silencing in VSMC attenuated proliferation and migration, while in EC these two cell functions appeared unaltered [114]. This of course does not preclude, that other EC functions may be affected by MARCKS silencing that could be relevant for vascular injury remodeling.

Conceptually an intriguing group of target genes are inflammatory cytokines and extracellular matrix proteins that are secreted by both, EC and VSMC. Arguably, silencing these targets in both cell fractions may prove beneficial.

## Translational potential and steps needed to get there

Establishing methods to reliably transfect specific cells of the vascular wall in a controlled and sustained fashion could have wide ranging clinical benefits. Naturally, atherosclerosis, aneurysmal disease, and intimal hyperplasia come to mind as primary disease entities. However, there are a variety of other clinical scenarios in which transient modulation of local vascular biology could be of great benefit. For instance it would be highly desirable to be able to increase endothelial barrier function in the setting of systemic inflammatory response syndrome and/or sepsis. Excessive vascular permeability and capillary leak lead to local edema with subsequent tissue hypoxia and acidosis. Pulmonary hypertension is another major cardiovascular condition that may be amenable to RNAi therapy [63, 115].

Taken together, the medical community would greatly benefit from the translation of the discussed pre-clinical studies to tangible medical therapies. Based on the existing data it appears that EC and macrophages currently might be the most promising target cells with regards to cell specific RNAi delivery. Both cell-types possess characteristic surface markers that could serve as receptors/ligands for cell specific RNAi delivery. Specific next steps towards clinical application also have to involve the transition from rodent to large animal models. While these are costly, they allow crucial insight in how to address larger vessel structures, circulating blood volumes, and flow patterns, more consistent with humans.

It seems pertinent to further optimize delivery of RNAi and develop methods for controlled sustained release. For a single local application, ligand-coupled nanoparticles containing RNAi or directly ligand-conjugated RNAi in combination with transcatheter administration might be a reasonable option. For systemic administrations as in the case of atherosclerosis, it might be useful to deploy receptor/ligand-RNAi constructs aimed at epitopes expressed in vascular lesions to direct RNAi preferentially to sites of high disease burden. Advances in RNAi structure design such as LNA have already yielded increased protection from enzymatic degradation and thereby increasing RNAi serum half-life [116]. Advances in technology to protect RNAi from non-specific tissue uptake (e.g. liver) are necessary to make systemic RNAi therapy more efficacious. Propensity of macrophages to take up RNAi complexes may in fact aid in a therapeutic approach for atherosclerosis. Thus, modulation of macrophage activity appears to be a promising approach as discussed above.

For sustained RNAi release approaches, perivascular and RNAi delivery from implantable cardiovascular devices may prove successful. As discussed before, several biologically compatible polymers exist that could serve as a depot for RNAi. Trapping RNAi in biodegradable materials with programmable degradation/release patterns needs to be further explored; specifically for cardiovascular applications.

In the opinion of the authors, it is important to first establish RNAi as a safe and programmable tool to modulate biological processes. As we start to understand RNAi-related challenges such as sequestration in liver, lung and macrophages, it might be judicious to first investigate the effects of RNAi in the lung vasculature and macrophages. If the desired results are achieved, increased resources could be dedicated to tackle other challenges relating to RNAi delivery to the vascular wall.

## Conclusions

As the understanding of vascular physiology and disease expanded over the past decades, it has become evident that significant loco-regional differences in vascular cell biology exist. Likewise, the technologies around RNAinterference have become increasingly sophisticated. However, anatomical barriers such as those found in large human blood vessels have not been adequately addressed. Also, off-site effects of RNAi therapy adjuncts can be significant and have to be carefully considered. Moreover, the *‘correct’* target gene choice for the individual vascular disease has yet to be made and it may be required to silence multiple genes in a synergistic fashion in order to achieve clinically relevant results. The reliable translation of promising in vitro or rodent animal data to large animal or human studies still has to occur. While multiple preclinical studies reported encouraging results of RNAi delivery to the vascular wall, it remains to be seen if a single target can be sufficient to the achieve clinically desirable changes in the injured vascular wall in humans. As discussed, the only existing clinical trial of vein graft treatment with oligonucleotides (PREVENT III) targeting E2F has failed to show clinically relevant results [26, 27]. While i.v. RNAinterference has been shown to be feasible for some organs; cell-specific, transmural vascular siRNA or miRNA delivery remains an unmet challenge. Off-target effects of RNAi and non-specific effects of transfection agents can significantly alter expression of a broad range of genes, which in turn could adversely influence the RNAi therapy results [29]. Successful therapies for vascular diseases may require the collaboration of an interdisciplinary team of geneticists, vascular biologists, chemical engineers and clinicians. Given the great burden that cardiovascular diseases inflict upon society, it is imperative to find new treatment opportunities. RNAi may be a technology that, when customized appropriately, could help ease that disease burden in the future.

## Abbreviations

AA: aortic aneurysm
ATH: atherosclerosis
BM: basal membrane
EC: endothelial cells
ICAM1: intercellular adhesion molecule 1
MARKS: myristoylated alanine rich protein kinase C substrate
miR: microRNA
PEG: polyethylene glycol
PEI: polyethylenimine
RISC: RNAi induced silencing complex
RNAi: RNA interference
siRNA: short interfering RNA
SMC: smooth muscle cells
SV-VSMC: saphenous vein vascular smooth muscle cells
SVV: survivin
TGF-beta: transforming growth factor beta
TSP-2: thrombospondin 2
VEGF: vascular endothelial growth factor
VEGF-R: vascular endothelial growth factor receptor
VCAM-1: vascular cell adhesion molecule 1
VSMC: vascular smooth muscle cells
